# Regional conditions determine thresholds of accelerated Antarctic basal melt in climate projection

**DOI:** 10.1038/s41558-025-02306-0

**Published:** 2025-04-10

**Authors:** Pengyang Song, Patrick Scholz, Gregor Knorr, Dmitry Sidorenko, Ralph Timmermann, Gerrit Lohmann

**Affiliations:** 1 Alfred Wegener Institute, Helmholtz Centre for Polar and Marine Research, Bremerhaven, Germany; 2 MARUM–Centre for Marine Environmental Sciences, University of Bremen, Bremen, Germany

**Keywords:** Climate and Earth system modelling, Physical oceanography

## Abstract

Antarctic basal melt is crucial for the future evolution of the Antarctic ice sheet and ocean circulation. However, few Earth system models explicitly simulate ice-shelf cavities. Here, using an Earth system model with interactive Antarctic ice-shelf cavities, we show that regional hydrography and topography determine a cavity tipping point. The Filchner–Ronne ice-shelf cavity will encounter such a tipping point with abrupt warm-water intrusion, rapid basal melt increase and massive freshwater release in response to increasing CO_2_ levels within this century. Conversely, the Ross Ice Shelf shows a more gradual response. Our results also suggest that previous ice-sheet modelling overestimated future ice-shelf melt, highlighting the need for comprehensive Earth system models with interactive ice-sheet dynamics and cavities for better climate projections.

## Abstract (web summary)

Melting from below is crucial for the future evolution of Antarctic ice shelves. Here the authors use an Earth system model with explicit simulations of ice-shelf cavities to show how regional hydrography and topography determine when an ice shelf will undergo rapid melting.

## Main

Previous studies have suggested that the West Antarctic Ice Sheet would collapse in a warming climate^1–3^, causing global sea level rise and large-scale ocean circulation changes. The Antarctic ice sheet (AIS) undergoes complex interactions, including surface mass balance, ice-shelf basal melting, ice-shelf calving, glacial isostatic adjustment and subglacial hydrology^4^. Among these processes, ice-shelf basal melting plays a critical role in grounding line position, ice-shelf buttressing, iceberg calving and ice flow towards the ocean.

Ice-shelf basal melting remains a major uncertainty in climate projections. Its impact extends to the Antarctic bottom water (AABW) formation^5–7^ and long-term carbon sequestration in the Southern Ocean^8^. Additionally, it may promote Antarctic sea-ice formation, mitigating anthropogenic warming through the ice–albedo effect^5,9–11^. Freshwater experiments are a major approach to discuss the influence of future ice-shelf basal melting^12^. However, this approach has several weaknesses. The projected freshwater flux comes from ice-sheet modelling, but ice-sheet models treat basal melt crudely^13^. The freshwater release occurs at the calving front rather than the actual ice-shelf–ocean interface. Most freshwater experiments prescribe freshwater forcing in a spatially uniform pattern. The latent heat through basal melt is usually ignored, causing a non-conserved ocean heat budget.

Ice-shelf cavity studies provide insights into projecting ice-shelf basal melting. By explicitly simulating these cavities, previous research revealed both the spatial pattern of basal melt rates and potential tipping points of large cavities. The Filchner–Ronne ice-shelf (FRIS) cavity is suggested to cross a tipping point under warming conditions^14–20^. The tipping point occurs when the dense shelf water (DSW) in the cavity becomes lighter than the modified warm deep water (mWDW), resulting in a flooding of mWDW into the cavity, a redirection of coastal currents and a drastic increase in basal melt. Though receiving less research focus than the FRIS cavity, the Ross ice-shelf (RIS) cavity is also suggested to cross a similar tipping point under warming conditions^20,21^. On a circum-Antarctic scale, studies reveal incoherent responses of Antarctic ice shelves to future climates^22^, emphasizing the critical role of sea-ice production^23^ and the sensitivity of basal melt to different climate states^24^.

Most ice-cavity research has focused on how ice-shelf cavities would respond to a warming climate. However, their role in the Earth system remains unclear. Here, we implement Antarctic ice-shelf cavities into an Earth system model (ESM) and conduct simulations following the Coupled Model Intercomparison Project Phase 6 (CMIP6) protocol^25,26^. Our focus is on Antarctic basal melt response and feedback to future climate. Simulations begin with a 750-year pre-industrial spin-up, followed by historical (HIST) and four Shared Socioeconomic Pathway scenario (SSP126, SSP245, SSP370 and SSP585) simulations^27^. We explore ice-cavity effects on the climate system through two experiment sets: one with ice-shelf cavities (ICE runs) and another with a traditional ocean set-up excluding cavities (CORE runs). Notably, iceberg melting, accounting for approximately 45% of current AIS mass loss^28^, is not included owing to its large uncertainty in projections^29^.

## Projections of Antarctic basal melt

**Fig. 1:**
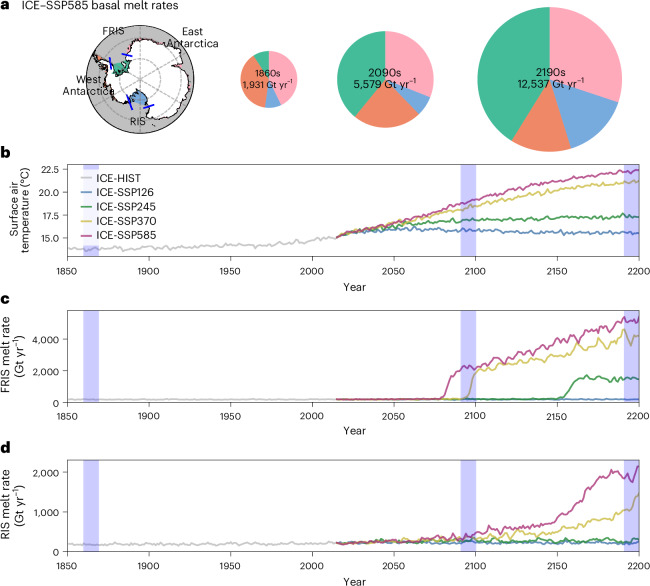
Projected Antarctic ice-shelf basal melt. **a**, Basal melt rates under the SSP585 scenario, showing separation between ice shelves (FRIS, East Antarctica, RIS and West Antarctica). The pie charts display basal melt rates and proportions for different periods, indicated in light blue in **b**–**d**. **b**, The global mean surface air temperature in HIST-to-SSP runs. **c**,**d**, The basal melt rate for FRIS (**c**) and RIS (**d**) in HIST-to-SSP runs.

Our results show that, under the high-emission SSP585 scenario, the total basal melt rate of Antarctic ice shelves increases by a factor of 2.9 by 2100 and 6.5 by 2200, relative to the pre-industrial condition with a melt rate of 1,931 Gt yr^−1^ (Fig. 1a). Notable increases can be observed for FRIS and RIS with basal melt rates rising from 178.6 to 5,160.3 Gt yr^−1^ between the 1860s and 2190s for the former, and from 180.6 to 1,892.4 Gt yr^−1^ between the 1860s and 2190s for the latter (Fig. 1c,d). The relative contribution of these two large ice shelves, known now as cold-water ice shelves, to the total basal mass loss increases from 18.6% in the 1860s to 45.8% in the 2090s and 56.3% in the 2190s.

The response of FRIS and RIS to future warming differs between the two ice shelves and between the four scenarios. RIS shows accelerating basal melt rates triggered by rising global temperatures under the SSP585 and SSP370 scenarios, while basal melt rates stay close to the pre-industrial conditions under the SSP245 and SSP126 scenarios. In contrast, FRIS shows abrupt increases in basal melt rates within a short period in three of the four scenarios, namely, in the 2080s, 2090s and 2150s for SSP585, SSP370 and SSP245, respectively. Before these tipping points, the basal melt rates are nearly unchanged, regardless of the global temperature increase (Fig. 1b). The transition is completed in about 10 years, with the basal melt rates increasing by a factor of 10. After the tipping points, the FRIS basal melt rates continue to increase in scenarios SSP585 and SSP370 while remaining largely constant in SSP245. Ensemble simulations under the SSP585 scenario confirm the significance of FRIS crossing a tipping point in the late twenty-first century, with timing subject to minor variations of 10 years (Supplementary Fig. 2e). Our ensemble results indicate that the simulated Antarctic basal melt rates exhibit minimal sensitivity to variations in initial conditions arising from internal variability.

## Isolated cavity and connected cavity

**Fig. 2:**
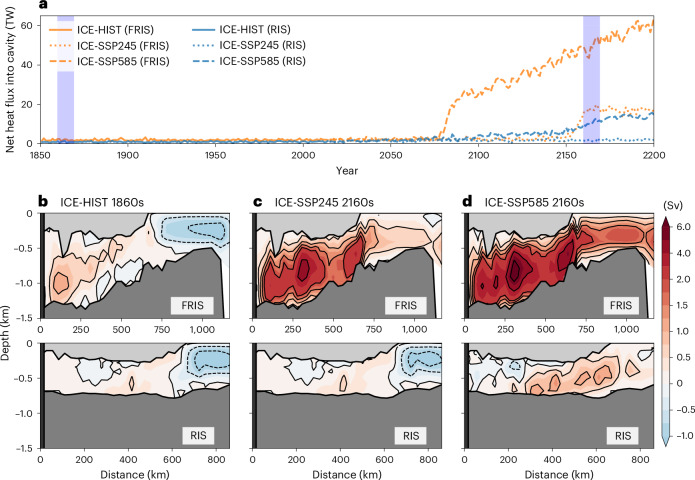
Ice-cavity overturning circulation. **a**, Time series of net heat flux into FRIS and RIS cavities from HIST, SSP245 and SSP585 runs. **b**–**d**, Overturning stream functions in FRIS (top) and RIS (bottom) cavities for ICE-HIST 1860s (**b**), ICE-SSP245 2160s (**c**) and ICE-SSP585 2160s (**d**), indicated in light blue in **a**. Red with solid contour lines indicates clockwise circulation, and blue with dashed contour lines indicates counterclockwise circulation. The contours and the colour bar are not uniformly spaced. TW, terawatt.

In a warming climate, the increased basal melt rates for FRIS and RIS result from increased heat fluxes into their cavities (Fig. 2a). These heat fluxes are linked to warm-water intrusion. The cavities exhibit distinct ‘cold’ and ‘warm’ modes based on overturning patterns. The cold-mode cavity has weak cavity overturning circulation, limited water exchange with the open ocean and a coastal downwelling cell near the ice-shelf front (Fig. 2b). Conversely, the warm-mode cavity features strong overturning, substantial water exchange and the absence of the coastal downwelling cell (Fig. 2d). The cold–warm shift reflects a reorganization of circulation and water-mass formation. In the cold mode, DSW forms at the ice-shelf front through sea-ice production, feeding the cavity water and blocking mWDW flow. In the warm mode, owing to reduced sea-ice production and DSW formation, mWDW rises to the continental shelf, intrudes into the cavity and discharges ice-shelf meltwater. Essentially, the shift is determined by the wrestling between DSW formation and mWDW intrusion. This mechanism aligns with previous modelling studies^14–20^.

**Fig. 3:**
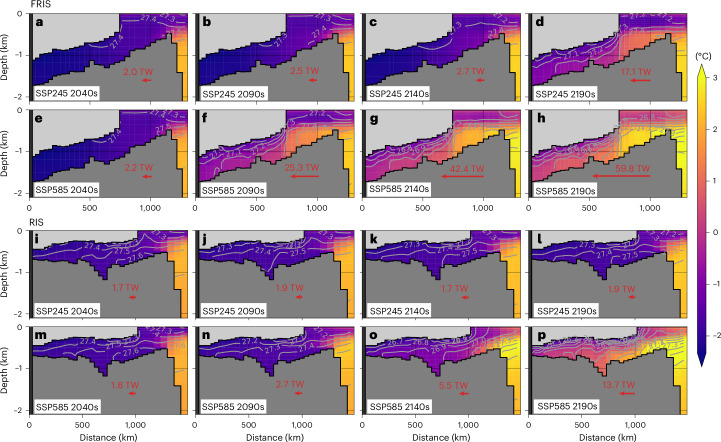
Warm-water intrusion in ice-shelf cavities. **a**–**p**, The evolution of potential temperature (colours) and potential density (contour lines) along sections in the Filchner trough (**a**–**h**) and Ross embayment (**i**–**p**) for SSP245 2040s (**a** and **i**), 2090s (**b** and **j**), 2140s (**c** and **k**) and 2190s (**d** and **l**) and SSP585 2040s (**e** and **m**), 2090s (**f** and **n**), 2140s (**g** and **o**) and 2190s (**h** and **p**). The coloured text and arrows indicate net heat flux into the FRIS or RIS cavity. Section locations are shown in Extended Data Fig. 1. TW, terawatt.

However, during the cold–warm shift, FRIS and RIS exhibit distinct behaviour in basal melt rates (Fig. 1c,d) and heat fluxes into their cavities (Fig. 2a). The FRIS cavity undergoes a rapid regime shift, while the RIS cavity experiences a slow displacement process. This difference can be explained by cross-section plots (Fig. 3). Initially, in the 2040s, the FRIS cavity is dynamically isolated from the open ocean, whereas the RIS cavity is not. Specifically, the FRIS cavity contains homogeneous cold and dense water during cold modes (Fig. 3a,e), while the RIS cavity contains stratified water with isopycnals connected to the open ocean (Fig. 3i,m). Geostrophic currents align with these isopycnal surfaces, allowing the stratified RIS cavity water to receive signals from the open ocean, while the homogeneous FRIS cavity water has minimal exchange through geostrophic currents.

**Extended Data Fig. 2:**
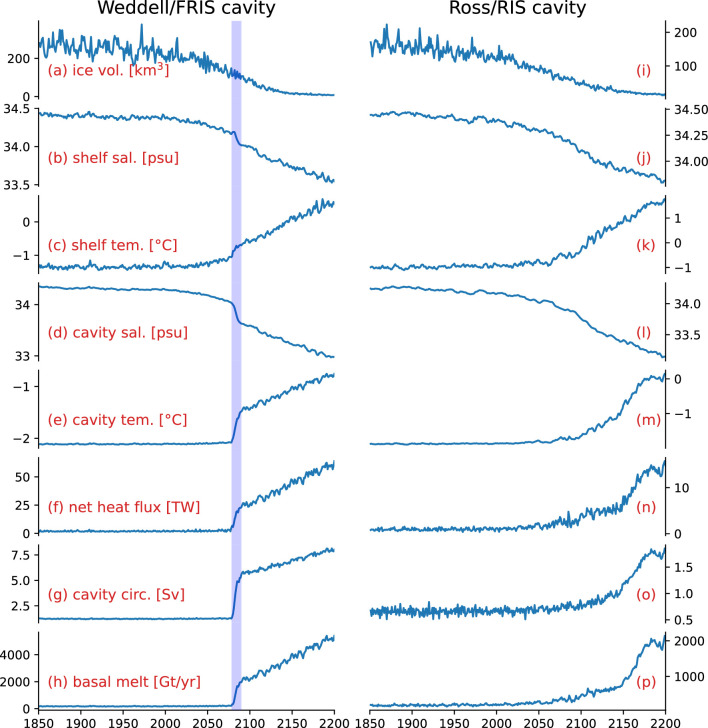
Annual-averaged time series of key indicators from the HIST-to-SSP585 runs. Indicators include (**a**) total sea-ice volume over the Weddell Sea continental shelf, (**b**) bottom salinity averaged over the Weddell Sea continental shelf, (**c**) bottom potential temperature averaged over the Weddell Sea continental shelf, (**d**) bottom salinity averaged in FRIS cavity, (**e**) bottom potential temperature averaged in FRIS cavity, (**f**) net heat flux into FRIS cavity, (**g**) overturning strength in FRIS cavity, (**h**) basal melt rate of FRIS. Panels (**i–p**) correspond to panels (**a–h**) but for the Ross Sea continental shelf/RIS cavity. The Weddell Sea continental shelf area is the open ocean in 62 °W–20 °W, south of 70 °S and shallower than 1000 m; the Ross Sea continental shelf area is the open ocean in 160 °E–150 °W, south of 72 °S and shallower than 1000 m. Light blue shading indicates the tipping point of FRIS cavity in ICE-SSP585 simulation.

Our results reveal a critical threshold for the FRIS cavity but not for the RIS cavity. Isopycnals act as an invisible gate, isolating the FRIS cavity water from the open ocean. As sea-ice formation decreases in a warming climate, the cavity water becomes fresher and more buoyant owing to a lack of salt supply. Once the cavity water becomes lighter than mWDW, mWDW invades the cavity (Extended Data Fig. 2a–h), and the invisible gate vanishes. In contrast, as with most other ice-shelf cavities in our simulations (Supplementary Fig. 1), the RIS cavity does not exhibit a tipping point because the stratification and isopycnals maintain a connected state initially, preventing a regime shift from isolated to connected states (Extended Data Fig. 2i–p). After crossing the tipping point, the FRIS cavity becomes ‘connected’, similar to the RIS cavity, whose basal melt rates are primarily influenced by mWDW entering cavities through deep channels in bottom topography. Our results show no substantial increase in mWDW temperature (open-ocean shelf–slope areas) under the SSP245 scenario (Fig. 3), explaining the nearly unchanged basal melt rates of RIS (all time) and the tipped FRIS (after the 2160s) in the ICE-SSP245 simulation.

In addition, the differences in cavity geometry and ocean bathymetry between FRIS and RIS should not be ignored. First, the difference in ice-shelf thickness determines the water exchange between the cavities and the open ocean^30^. FRIS has a thickness of about 400 m at the ice-shelf front. According to the conservation of potential vorticity, the topographic barrier forces a barotropic blocking pattern in the circulation and hydrography (Figs. 2c and 3d). Compared with FRIS, the RIS front is thinner (about 200 m) and the topographic barrier is weaker. Second, the slope of the ice-shelf base determines the overturning strength in ice-shelf cavities. As previous research^31–33^ suggested, the RIS cavity naturally features weaker overturning strength than the FRIS cavity because it largely lacks the ‘ice pump’ originating from the tilting ice-shelf base. Third, the difference in ocean bathymetry partly determines the response time of cavities to signals from the open ocean. The bathymetry of the FRIS cavity is sloping, while the bathymetry of the RIS cavity is flat. The consequence is that once the mWDW intrudes into the FRIS cavity, gravitational force drives the water to the grounding line. In the RIS cavity, however, a warming signal from the ice-shelf front would take longer to travel to the grounding line because of the flat bathymetry.

Our results show that from a dynamic perspective, homogeneous or stratified cavity water mainly determines whether the cavity will experience rapid or slow shifting in a warming climate, respectively. In contrast to our results, ref. ^21^ showed a rapid-shifting process for the RIS cavity. We attribute this difference to the homogeneous and dense RIS cavity water shown in their simulation. Therefore, the projection of cavity tipping points largely depends on simulating the evolution of isopycnal surfaces, which is related to the oceanic and atmospheric states. Indeed, previous numerical studies reveal that the possible FRIS cavity tipping in a projection framework depends on the prescribed atmospheric forcing^15,18–20,23,34,35^. In addition in ref. ^36^, an unrealistic tipping of the FRIS cavity was shown during the pre-industrial spin-up when the eddy diffusion in Gent–McWilliams parameterization^37^ is set spatially constant instead of spatially varying. With a stronger eddy-induced mixing, their model demonstrates flatter isopycnal surfaces, which results in severe cross-shelf transport and warm-water intrusion during the spin-up. Considering the substantial uncertainty arising from divergent modelling studies and disparities between models and observational data (Supplementary Table 1), there is a compelling need for model intercomparison initiatives incorporating ice-shelf cavities within state-of-the-art ESMs.

## Climatic feedback of ice cavities

**Fig. 4:**
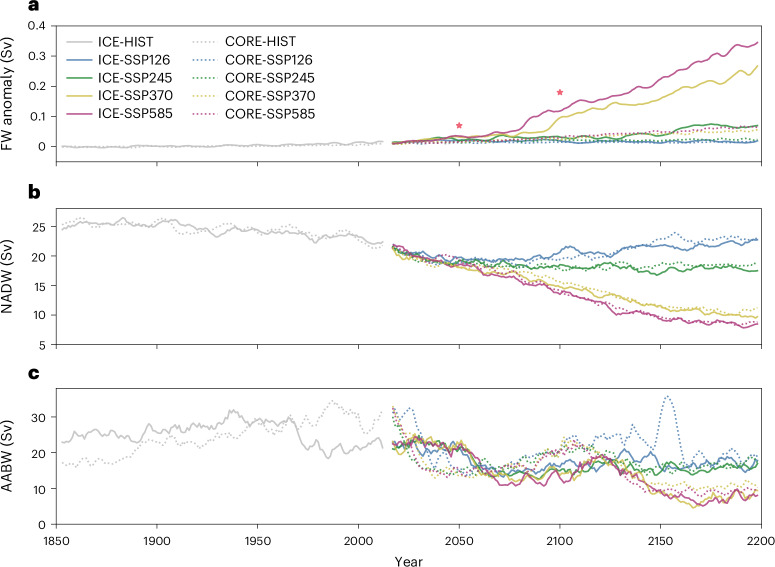
Climate projections with and without ice-shelf cavities. **a**–**c**, Time series of Antarctic freshwater anomaly (surface run-off for CORE runs and ice-shelf meltwater for ICE runs) (**a**), NADW formation (**b**) and AABW formation (**c**) for HIST and SSP runs. The anomaly values are relative to the pre-industrial state (1851–1900 average). The stars in **a** denote the extreme-scenario Antarctic basal melt anomalies for 2050 and 2100 from ref. ^44^ and ref. ^2^, respectively. The NADW formation is estimated from the positive maximum of the Atlantic MOC stream function (40 °N–80 °N). The AABW formation is estimated from the negative maximum of the global MOC stream function (80 °S–50 °S). All time series apply a 5-year moving average.

**Extended Data Fig. 3:**
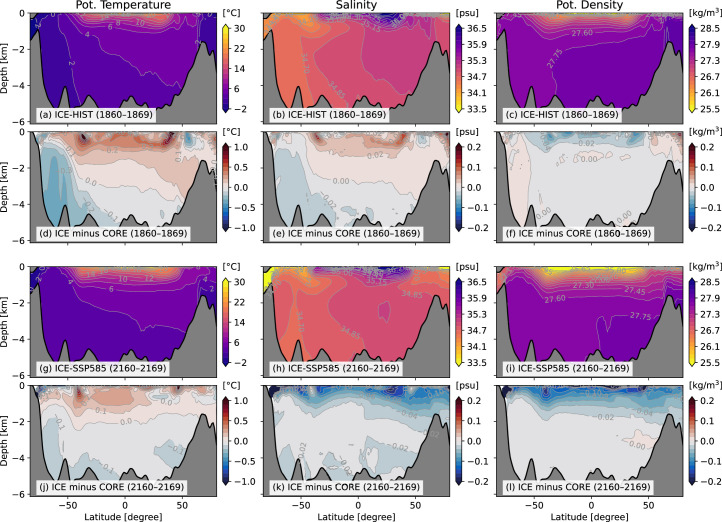
Stratification in the Atlantic basin from ICE and CORE runs. (**a–c**) Section plots of hydrography (potential temperature, salinity and potential density) in the Atlantic basin from ICE-HIST (1860s). (**d–f**) Section plots from CORE-HIST (1860s) but are shown by the differences between ICE and CORE runs. (**g–i,j–l**) The same as (**a–c**) and (**d–f**) but for the 2160s under the SSP585 scenario.

To isolate the effect of ice-shelf cavities on climate projections, we compare the large-scale circulation between ICE and CORE runs. Compared with CORE runs, ICE runs not only extend the model domain to ice-shelf cavities, but also release freshwater differently. In ICE runs, Antarctic freshwater flux results from explicitly calculating basal melt rates. In contrast, CORE runs adopt a traditional treatment, assuming that AIS is in balance. Thus, excess precipitation on AIS is delivered directly into the adjacent coasts as surface run-off. The most noticeable difference between ICE and CORE runs is the accelerated Antarctic basal melt observed in ICE runs under high-emission scenarios (Fig. 4a). By 2200, Antarctic freshwater release from CORE-SSP runs exhibit small sensitivity across climate states (anomalies ranging from 0.02 Sverdrup (Sv) in SSP126 to 0.06 Sv in SSP585). In contrast, Antarctic ice-shelf meltwater from ICE-SSP runs exhibit notable sensitivity (anomalies ranging from 0.02 Sv in SSP126 to 0.34 Sv in SSP585) due to explicitly simulating ice-shelf basal melting. Both ICE-SSP and CORE-SSP runs indicate declining North Atlantic deep water (NADW) formation under a warming climate (Fig. 4b). Interestingly, the differences between ICE and CORE within the same scenario are not substantial, suggesting that the Antarctic ice-shelf meltwater has a minor impact on NADW formation^38^. Though massive Antarctic ice-shelf meltwater freshens the upper Atlantic Ocean, it does not create a meridional density gradient in the Atlantic basin (Extended Data Fig. 3l), a crucial factor controlling NADW strength^39^. Regarding AABW formation (Fig. 4c), considerable differences in strength and decadal-scale trends emerge between ICE and CORE runs. Both ICE and CORE runs show declining AABW formation under a warming climate. However, ensemble members exhibit considerable uncertainties in AABW projections (Supplementary Fig. 2c,d). This is because the coupled model features a centennial-scale natural variability in Southern Ocean convection^40^. Therefore, different branch-off years of the individual ensemble members lead to different phases of Southern Ocean convection (Supplementary Fig. 3).

**Fig. 5:**
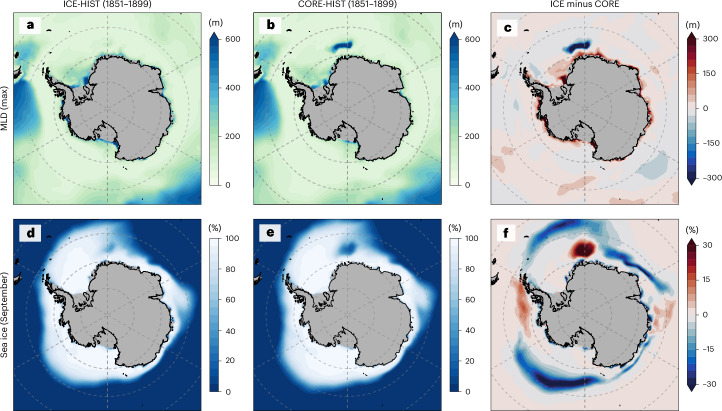
Southern Ocean mixed-layer depth and sea-ice concentration. **a**–**c**, The climatological maximum of monthly mixed-layer depth in ICE-HIST (**a**) and CORE-HIST (**b**) simulations and the difference between ICE-HIST and CORE-HIST (**c**). **d**–**f**, The September sea-ice concentration in ICE-HIST (**d**) and CORE-HIST (**e**) simulations and the difference between ICE-HIST and CORE-HIST (**f**). Mixed-layer depth (MLD) is defined by the 0.03 kg m^*−*3^ density difference criterion. The results are averaged from 1851 to 1899.

**Extended Data Fig. 4:**
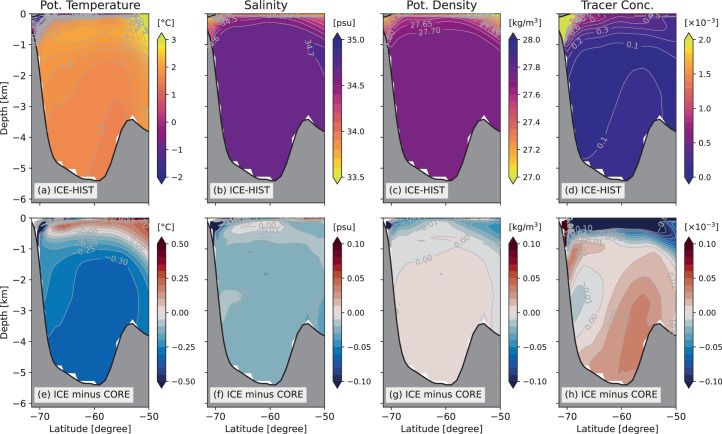
Stratification in the Weddell sector from ICE-HIST and CORE-HIST. Section plots show (**a**) potential temperature, (**b**) salinity, (**c**) potential density and (**d**) passive tracer concentration along the 0 ° meridian in the Southern Ocean. Panels (**e–h**) show differences between ICE and CORE. The meridional section is zonally averaged from 10 °W to 10 °E, with a 1 ° meridional bin. Results are based on ICE-HIST and CORE-HIST from 1851 to 1899. The release of the passive tracer is explained in Methods.

In a pre-industrial climate, considering ice-shelf cavities rectifies AABW formation sites in the model (Fig. 5). Compared with CORE-HIST, ICE-HIST exhibits shallower mixed layer in the open ocean but deeper on the Antarctic continental shelf. Additionally, ICE-HIST shows less pronounced open-ocean polynyas in the Weddell Sea. These changes suggest that compared with CORE-HIST, ICE-HIST features more dense water formed on the continental shelf owing to brine rejection and less dense water formed in open-ocean polynyas owing to surface cooling (Supplementary Figs. 4 and 5). Crucially, ICE runs considerably reduce the intensity and depth of open-ocean deep convection, which is spuriously high in the CORE runs and many CMIP6 models^41^. This is because of three reasons as follows: (1) latent heat is considered during ice-shelf basal melting. Therefore, supercooled ice-shelf meltwater contributes to AABW and favours AABW sinking at continental shelf–slopes in ICE runs (Extended Data Fig. 4h); (2) the sinking of ice-shelf meltwater results in a cooler deep Southern Ocean (Extended Data Fig. 4e), reducing deep ocean heat accumulation and upwards heat flux, thus mitigating open ocean polynyas, which solves similar problems in previous modelling studies^40,42,43^; and (3) ice-shelf meltwater is released at the ice-shelf–ocean interface. It also mitigates open-ocean polynyas by impacting subsurface stratification and blocking upwards heat flux (Extended Data Fig. 4g).

In a warmer climate, an enhanced hydrological cycle (that is, increased precipitation over the Southern Ocean) and a reduced Antarctic sea-ice formation mainly explain the weaker AABW formation in both experiments. In addition, the disappearance of open-ocean polynyas causes the reduction of AABW formation in CORE runs, while increased ice-shelf basal melt and the related freshening of water contribute to the reduction of AABW formation in ICE runs. Notably, as sea-ice extent shrinks to the Antarctic continental shelf in a warmer climate, surface cooling (that is, upwards air–sea heat flux) contributes more to DSW formation than in a colder climate, when extensive sea ice isolates the ocean from the atmosphere (Supplementary Fig. 6). The increase in DSW formation owing to surface cooling explains the slight increase of AABW formation around 2100 in both ICE and CORE simulations, for example, a slightly higher AABW formation in the 2110s compared with the 2070s in ICE-SSP585 (Fig. 4c).

**Extended Data Fig. 5:**
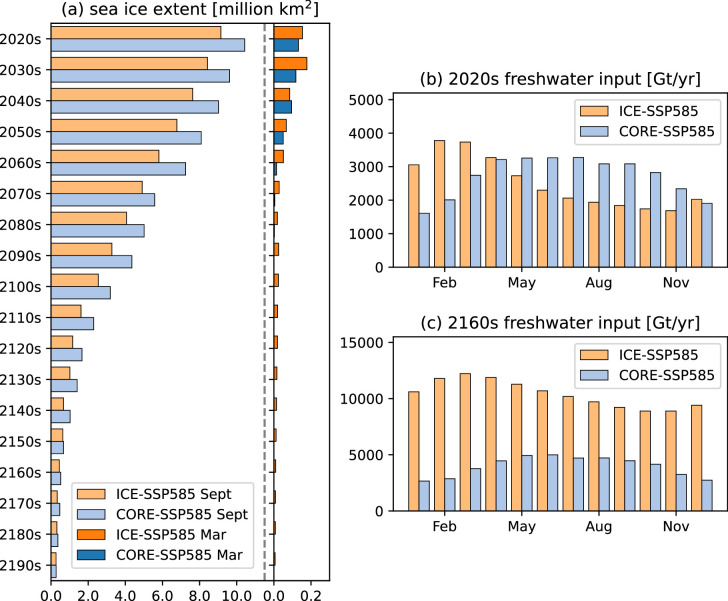
Southern Ocean sea-ice extent and Antarctic freshwater release. (**a**) Decadal-averaged September/March Antarctic sea-ice extent in ICE and CORE runs. Note that the horizontal axis for March is scaled by a factor of 10. (**b,c**) Multi-year monthly averaged Antarctic freshwater release in the 2020s and 2160s. Results come from ICESSP585 and CORE-SSP585.

Furthermore, considering hydrosphere–cryosphere interaction in our ESM reveals two previously unnoticed processes. First, our ice-cavity simulations demonstrate a delayed basal melt response to climate warming. Our results show a 0.12 Sv Antarctic ice-shelf meltwater anomaly by 2100 under the SSP585 scenario, similar to ref. ^21^ and larger than ref. ^23^. Ice-sheet modelling studies^2,44^ predicted higher basal melt rates under the extreme emission scenario (0.07 Sv until 2050 and 0.18 Sv until 2100; Fig. 4a). The deviation arises from neglecting ice-cavity processes in ice-sheet models. As the propagation of warming signals in ice-shelf cavities takes time, ice-cavity simulations feature a lag between the open-ocean warming and the basal melt increasing, which can be indicated by the temperature decrease from continental shelf break to the grounding line (Fig. 3). Therefore, an ice-sheet model forced by the bottom temperature on continental shelf tends to overestimate basal melt rates. Second, ice-shelf meltwater exhibits a seasonal cycle, with peak melting in austral summer and minimal melting in austral winter. However, freshwater forcing experiments typically ignore this seasonality^10^. Compared with winter-released freshwater, summer-released freshwater is negligible in expanding sea-ice areas and reducing anthropogenic warming through the ice–albedo effect (Extended Data Fig. 5). Therefore, erasing the seasonality of freshwater forcing would overestimate winter-released freshwater and underestimate summer-released freshwater, while in total overestimating sea-ice formation.

## Concluding remarks

We introduce Antarctic ice-shelf cavities into the ocean component of a complex ESM. By showing the responses and feedbacks of the Antarctic ice-shelf cavities from a climate perspective, we suggest that the representation of the cryospheric feedback in an ESM is substantially improved when interactive ice sheets and their cavities are involved, and one can better estimate basal melt, grounding line retreat and sea level rise with such an ESM. Therefore, one future direction is to set up a fully coupled ESM with dynamic ice sheets, flexible cavity shapes and iceberg calving to study past, present and future climate changes. It is also important to acknowledge that our CMIP6-type simulation still employs relatively low horizontal and vertical resolutions. The representation of the Antarctic shelf–slope current system and the descent of AABW into the abyss in our study is not as accurate as in high-resolution studies^6,45–47^. Therefore, another future direction is to explore a high-resolution ocean set-up that includes ice-shelf cavities to gain a more comprehensive understanding of Southern Ocean dynamics.

## Methods

### Model description

In this work, we simulate Antarctic ice-shelf basal melt rates using the Alfred Wegener Institute Earth System Model (AWI-ESM2). AWI-ESM2 is a fully coupled ESM comprising the ocean–sea-ice model FESOM2 (ref. ^48^), the atmosphere model ECHAM6 (ref. ^49^) and the land surface model JSBACH^50,51^. As a submodel of ECHAM6, JSBACH provides interactive vegetation dynamics and hydrologic cycles. The coupled model was first introduced in ref. ^52^ and has been validated for both paleo and present climates^53–56^.

A standard configuration of AWI-ESM2 includes the following. The oceanic component FESOM2 applies a CORE-II mesh with 48 unevenly distributed layers in the vertical direction^57^. The atmospheric component ECHAM6 applies a T63 Gaussian grid and 47 layers based on a hybrid sigma–pressure coordinate. The land surface component JSBACH applies a T63 Gaussian grid with five soil layers to calculate the energy balance and thermal diffusion on land, and the subgrid scale heterogeneity in JSBACH is represented by 11 tiles in each grid box. The time step is 450 s for ECHAM6/JSBACH and 1,800 s for FESOM2. The coupling time step, representing the frequency of data exchange between the atmospheric and oceanic components, is 3,600 s.

Ice-shelf cavities are a feature of FESOM2 following the model in ref. ^58^. Similar to the ocean bathymetry, ice shelves provide a solid upper boundary in the ocean model. In the cavities, the ocean surface is the ice-shelf–ocean interface instead of the atmosphere–ocean interface. Therefore, all atmosphere–ocean interactions and sea-ice processes are replaced by the momentum, heat and salt flux at the ice-shelf base. The momentum flux considers frictional stress provided by a solid ice-shelf boundary, while the heat and salt fluxes are parameterized following previous studies^31,59^. Moreover, the heat and salt fluxes adopt velocity-dependent coefficients as in ref. ^60^. During the simulations, cavity geometry and ocean bathymetry are fixed.

FESOM2 supports an Arbitrary Lagrangian–Eulerian vertical coordinate^48,57^, which assembles different vertical coordinates in the same framework. A standard configuration selects a variant of the *z*-coordinate, namely, the *z*-star coordinate, where the total change in sea surface height is equally distributed over all water grids in the vertical, except the grid involving the ocean floor. When ice-shelf cavities are considered, cavity grids adopt an approximation of linear free surface, while open ocean grids remain full free surface. Under the condition of linear free surface, the volume of each grid cell in cavities is fixed, and the freshwater flux through ice-shelf–ocean interface is treated as a virtual salt flux.

This study aims to conduct long-term simulations of the multisphere complex Earth system. Owing to computing limitations, the mesh used does not explicitly resolve mesoscale eddies. Therefore, the Gent–McWilliams parameterization^37^ with Redi isoneutral diffusion^61^ is applied in ocean models. Particularly, we use an improved Gent–McWilliams scheme^62^ designed to better handle weakly stratified areas by solving a local vertical structure of eddy-induced transport with a one-dimensional boundary-value equation. In addition, the eddy-diffusion coefficient is adjusted by a horizontal damping scale on the basis of mesh resolution, preventing the overestimation of eddy-induced transport where the model can resolve large eddies with mesh sizes of up to ~15 km.

### Model meshes

The CORE-II mesh is a commonly used unstructured ocean mesh resolving different ocean scales in FESOM2 and AWI-ESM2. It is characterized by low resolution (1°) in mid-latitude areas and high resolution at coastal regions, the equator (0.33°) and north of 50 °N (25 km). However, the CORE-II mesh does not include the Antarctic ice-shelf cavities. To conduct a numerical study exploring areas under the Antarctic ice shelves, we use a mesh extension strategy. The mesh extension strategy keeps the existing CORE-II mesh while adding more triangles and nodes to the boundaries. Compared with creating an entirely new mesh, the mesh extension strategy limits all changes to the Antarctic area, thus avoiding the possibility that mesh differences in the far field also affect the results.

Originating from the CORE-II mesh, we first generate the CORE-MAX mesh, extending to the sea level lines under the AIS, that is, the intersection of the present sea level and Antarctic bedrock. Considering the purpose of the CORE-II mesh, which is to study long-term climate with a moderate computational cost, the resolution of the additional sectors covering the grounded ice-sheet and ice-shelf cavities is 16 km on average. The CORE-MAX mesh is not used in this study but is meant to serve as a mother mesh for further research on ocean responses to the future AIS evolution. In this work, we apply a child mesh, CORE-ICE, which is generated by dropping out the areas covered by grounded ice sheets in the mother mesh (CORE-MAX), similar to the strategy developed in ref. ^34^ for a coupled ice-sheet–ocean model with FESOM-1.4 as the ocean component. The ocean bathymetry and cavity geometry of AIS are derived from the RTopo-2 dataset^63^.

It should be noted that not only the ocean mesh but also the land–sea mask in the atmosphere model should be changed in a model configuration with Antarctic ice shelves. Since ice-shelf areas provide a glacier-like boundary condition to the atmosphere, the land–sea mask file for ECHAM6/JSBACH was also modified accordingly.

### Simulation description

The ocean component is initialized from the Polar Science Center Hydrographic Climatology^64^, and the hydrography inside ice-shelf cavities is initialized via extrapolation. We first conduct two 1,000-year spin-up simulations under a pre-industrial climate with CORE-II mesh and CORE-ICE mesh, respectively. The simulations are named CORE-PI and ICE-PI. From the pre-industrial simulations, the HIST simulations, CORE-HIST and ICE-HIST, are branched off at the 750th model year. CORE-HIST and ICE-HIST are integrated from 1851 to 2014. Then, from the end of the HIST simulations, each simulation bifurcates into four SSP scenarios, ranging from 2015 to 2200. Though a standard SSP scenario run ends in the year 2100, we extend them to 2200 to observe more effects of ice-shelf cavities. For simulation between 2100 and 2200, the anthropogenic forcing file related to land use is extrapolated using the values in 2100, while for greenhouse gases, we apply values from ref. ^27^. All experiments are conducted with the AWI-developed ESM tools^65^.

Antarctic freshwater input should be treated carefully in simulations. CORE runs apply a traditional way to treat Antarctic freshwater input based on a mass balance of AIS. Specifically, CORE runs discharge excess precipitation on AIS to the nearest coastal ocean grid point as surface run-off. In contrast, ICE runs consider freshwater input as melting at the ice-shelf base, which is parameterized in FESOM2. To avoid redundancy of freshwater forcing, we shut down the Antarctic surface run-off in ICE runs. By default, both CORE and ICE runs apply volume and salt conservation over the global ocean. However, considering the accelerating ice-shelf basal melt during the HIST-to-SSP periods, we do not conserve the global volume and salt content in the ICE-HIST and ICE-SSP runs.

To assess the robustness of our model results, we apply ensemble simulations for both ICE and CORE runs during the HIST-to-SSP585 scenario period. Each ensemble contains nine members, characterized by different branch-off years from the corresponding spin-up simulation. Nine branch-off points range from year 650 to 850 with an interval of 25 years. The approach of creating an initial-condition ensemble is aimed to represent the influence of internal variability on our research focus^25^. The nine ensemble members are in order named ICE/CORE-ens1 to ICE/CORE-ens9. Note that ICE/CORE-ens5 is identical to the ICE/CORE-HIST and ICE/CORE-SSP585 simulations.

In addition, to investigate the influence of Antarctic freshwater input on water-mass distribution in the Southern Ocean, we add passive tracers in the HIST and SSP scenario runs. We assume that the freshwater released from Antarctica contains passive tracers whose concentration is unity. In CORE runs, the passive tracers are released along with surface run-off from Antarctica, while in ICE runs, those are released along with Antarctic ice-shelf meltwater.

We should also mention that the ICE runs do not consider a changing cavity geometry, including ice-shelf thickness change, grounding-line retreat and ice-shelf calving. Iceberg melting is also not considered in this work.

### Density framework diagnosis

The density framework in FESOM2 is explained in refs. ^66,67^. The diagnostic module facilitates the calculation of meridional overturning circulation (MOC; Supplementary Fig. 1i) in a density coordinate, effectively addressing the limitation of representing MOC in a depth coordinate. Specifically, this approach rectifies the representation of the Deacon cell in the Southern Ocean^68^. In this work, the calculation of NADW/AABW formation is based on Atlantic/global MOC in a density coordinate.

**Extended Data Fig. 6:**
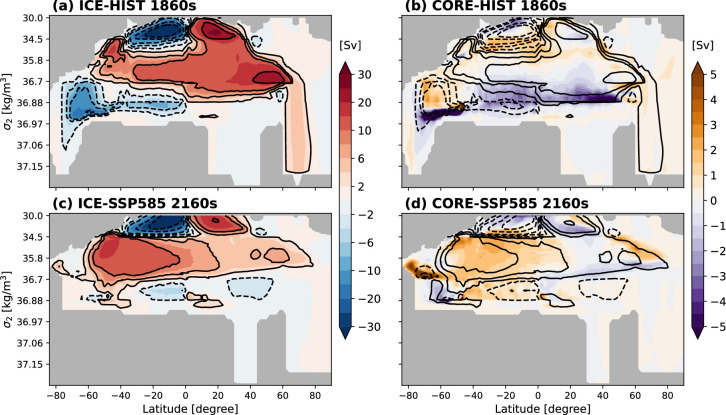
Global meridional overturning streamfunctions in density coordinates. (**a,c**) Overturning streamfunctions in ICE-HIST (1860s) and ICE-SSP585 (2160s). Red solid contours indicate clockwise circulation; blue dashed contours indicate counterclockwise circulation. (**b,d**) Differences in overturning streamfunctions between ICE and CORE. The contour lines show streamfunctions in CORE runs, but the colours show differences between ICE and CORE (ICE minus CORE). Density coordinate σ_2_ denotes potential density referenced to 2000 m depth. The contours and the colour bar for panels (a) and (c) are not uniformly spaced.

The global MOC in density coordinates intuitively illustrates water-mass formation in both pre-industrial and extreme warming states (Extended Data Fig. 6). Despite the decline of NADW and AABW formation in a warmer climate, differences in water properties between ICE and CORE runs are highlighted. For example, ICE-HIST demonstrates a denser and stronger AABW cell than CORE-HIST in the pre-industrial state (Extended Data Fig. 6b) owing to the contribution of ice-shelf meltwater in AABW. In addition, though massive ice-shelf meltwater in ICE-SSP585 does not result in a weaker NADW, its NADW cell is indeed shallower than CORE-SSP585 in density space (Extended Data Fig. 6d), which coincides with the fresher upper Atlantic basin (Extended Data Fig. 3l).

Furthermore, the model computes surface diapycnal transformation rates resulting from surface heat flux and surface salt flux. Therefore, the density MOC can be decomposed by different oceanic processes, including surface heat flux, surface salt flux and internal mixing^69^. The decomposition could provide a more detailed explanation of the AABW differences between ICE and CORE. For example, ICE-HIST features more AABW formation through brine rejection than CORE-HIST (Supplementary Fig. 4c). In contrast, CORE-HIST displays a deep cell formed owing to surface cooling, around 36.96 kg m^*−*3^, indicating the deep mixed water located at open-ocean polynyas (Supplementary Fig. 4f). This can be further indicated by surface water-mass transformation at individual density levels (Supplementary Fig. 5p).

A horizontal integration of surface water-mass formation rates over the Antarctic continental shelf represents DSW formation. In a warmer climate, the ice-shelf melting in ICE-SSP585 results in DSW formation moving to a shallower density level, a change that is minor in CORE-SSP585 (Supplementary Fig. 6). Notably, in a warmer climate, despite that an enhanced hydrological cycle (that is, increased precipitation over the Southern Ocean) and a reduced Antarctic sea-ice formation largely weaken DSW formation through brine rejection, DSW formation through surface cooling slightly increases. This is because the sea-ice extent shrinks to the Antarctic continental shelf, and upwards air–sea heat flux contributes more to DSW formation owing to a lack of sea-ice isolation.

### Model validation

Though AWI-ESM2 has been validated in many studies, this study examines the ice-cavity module in the ocean component FESOM2. In this section, we show the basal melt rates from both AWI-ESM2 and FESOM2 simulations and compare them with previous studies. The AWI-ESM2 simulation refers to the HIST period of the ICE ensemble mentioned above. The FESOM2 simulation applies the same model configuration as AWI-ESM2 but is an ocean-only simulation driven by the atmospheric forcing JRA55-do^70^ from 1958 to 2020. Note that ensemble simulations are not applied for the ocean-only set up.

Basal melt rates from our simulations are integrated over different areas, as shown in Supplementary Fig. 1i. These values are compared with those estimated from other studies via observation or modelling (Supplementary Table 1). Both the FESOM2 and AWI-ESM2 results presented in Supplementary Table 1 are averaged from model year 2001 to 2010. The basal melt rates and their uncertainties from AWI-ESM2 are given by the ensemble mean and 1 s.d.

Compared with the estimates of basal melt rates based on observational data, model studies reveal large uncertainty. Our FESOM2 simulation agrees well with the observational data regarding the total basal melt rate, but features a significantly lower basal melt rate for the Amundsen Sea (area G) and higher for the RIS (area F) and the Eastern Weddell region (area C). In contrast, the AWI-ESM2 simulation largely overestimates the basal melt rates in the Eastern Weddell region (area C), the Amery Ice Shelf (area D), RIS (area F) and the Bellingshausen Sea (area H). The defined areas are shown in Supplementary Fig. 1i.

The FESOM2 results suggest that the ice-cavity module can reproduce current basal melt rates for most Antarctic ice shelves. AWI-ESM2 exhibits a larger bias in basal melt rates than FESOM2 owing to its larger hydrographic bias in the ocean. A freely developing atmospheric component causes the coupled model to be less constrained and more biased than an ocean-only model forced by a prescribed atmospheric forcing field. This is commonly found in many other coupled simulations.

## Online content

Any methods, additional references, Nature Portfolio reporting summaries, source data, extended data, supplementary information, acknowledgements, peer review information; details of author contributions and competing interests; and statements of data and code availability are available at 10.1038/s41558-025-02306-0.

## Supplementary information

Supplementary Information Supplementary Table 1, Figs. 1–6 and References.

## Figures and Tables

**Extended Data Fig. 1:**
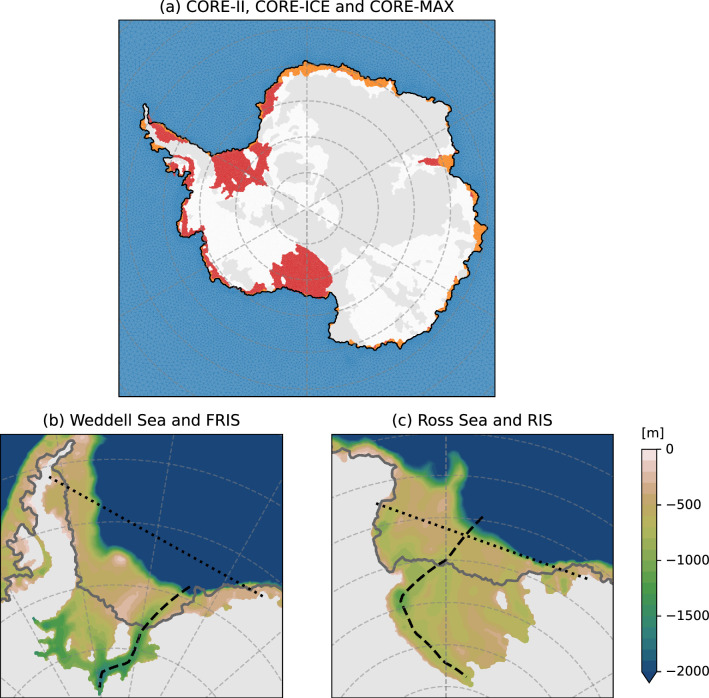
Model meshes and section locations. (**a**) Meshes: CORE-II (blue/orange), CORE-ICE (blue/orange/red) and CORE-MAX (except grey). Orange mesh represents open-ocean areas in CORE-II but ice shelf cavities in CORE-ICE. White mesh represents areas of grounded ice sheets. Black line shows the AIS extent in the model. (**b,c**) Colours show ocean bathymetry in the model. Solid grey lines represent the AIS extent, identical to the calving front for ice shelves. Dashed black lines indicate the section plot locations. Dotted black lines indicate the northern boundaries for calculating overturning streamfunctions in FRIS and RIS cavities. In ice-cavity streamfunction plots (Fig. 2), the x-axis is normal to the dotted black lines.

## Data Availability

The raw model output is not deposited into a public data repository due to its size. Essential intermediate results for reproducing the paper’s figures are available via Zenodo at https://zenodo.org/records/14186137 (ref. ^71^). Full raw model output is available upon request from the corresponding author.

## Code availability

Model source codes, configuration files, mesh files, post-processing and visualization codes are archived and available via Zenodo at https://zenodo.org/records/14186137 (ref. ^71^). Simulation results are available upon request from the corresponding author.
