# Supplementary material for: Regional conditions determine thresholds of accelerated Antarctic basal melt in climate projection
Source: Nat Clim Chang. 2025 Apr 10;15(5):521–7. doi: 10.1038/s41558-025-02306-0 (PMC12064438; doi:10.1038/s41558-025-02306-0)
Supplement: Supplementary file 1 — Supplementary Table 1, Figs. 1–6 and References. [file 41558_2025_2306_MOESM1_ESM.pdf]

nature climate change

Supplementary information https://doi.org/10.1038/s41558-025-02306-0

Regional conditions determine thresholds of accelerated Antarctic basal melt in climate projection

In the format provided by the authors and unedited

Contents

Model validation **2**

Projection of AIS basal melt by regions **3**

Ensemble simulation results **4**

Density framework diagnosis **6**

References for Supplementary Information **9**

Model validation

Supplementary Table S1 Comparison of Antarctic ice shelf basal melt rates in this study with observational and other numerical studies. Melt rates and uncertainties for AWI-ESM2 are shown as mean and one standard deviation of the ensemble members. Area A to H are indicated in Supplementary Fig. S1. Two observational studies [1, 2] show discrepancies between the sum of the individual estimates and the total given for the whole Antarctica, which can be attributed to the contribution of the many very small ice shelves in several sectors of the Antarctic coastline. Thus, the values shown in this table are proportionally upscaled so that the total corresponds with their estimates for the total Antarctica. In the table, “n/a”s mean the areas are not indicated in that numerical study, and numbers in round brackets represent incomplete estimations because small ice shelves are not counted in that numerical study. All values are in units of Gt/yr.

Study	A	B	C	D	E	F	G	H	total
Rignot (2013)	42	176	110	42	288	54	528	259	1499
Depoorter (2013)	20	56	151	50	339	40	517	281	1454
Adusumilli (2020) 1994–2018 avg.	113	81	161	46	210	80	369	205	1265
Paolo (2023)	42	60	76	31	179	28	435	173	1024
AWI-ESM2	93±8	182±6	429±13	253±11	289±7	213±6	416±32	497±39	2372±80
FESOM2	54	198	332	70	187	130	164	199	1335
Hellmer (2004)	38	120	n/a	18	n/a	180	n/a	n/a	907
Timmermann (2012)	48	138	n/a	174	n/a	260	n/a	n/a	1600
Kusahara (2013)	22	161	108	70	83	111	64	152	770
Schodlok (2016) 50-layer	14	793	212	68	220	190	813	558	2868
Schodlok (2016) 100-layer	95	108	186	75	88	167	670	345	1735
Mathiot (2017)	46	123	n/a	207	n/a	111	n/a	n/a	1456
Naughten (2018) MetROMS	21	46	(100)	91	(39)	54	(144)	(88)	642
Naughten (2018) FESOM-LR	37	114	(123)	71	(35)	95	(36)	(50)	586
Naughten (2018) FESOM-HR	58	115	(137)	71	(49)	112	(63)	(91)	739
Siahaan (2022)	n/a	52	n/a	n/a	n/a	58	n/a	n/a	982
Verfaillie (2022)	n/a	159	n/a	n/a	n/a	63	373	90	979

Projection of AIS basal melt by regions

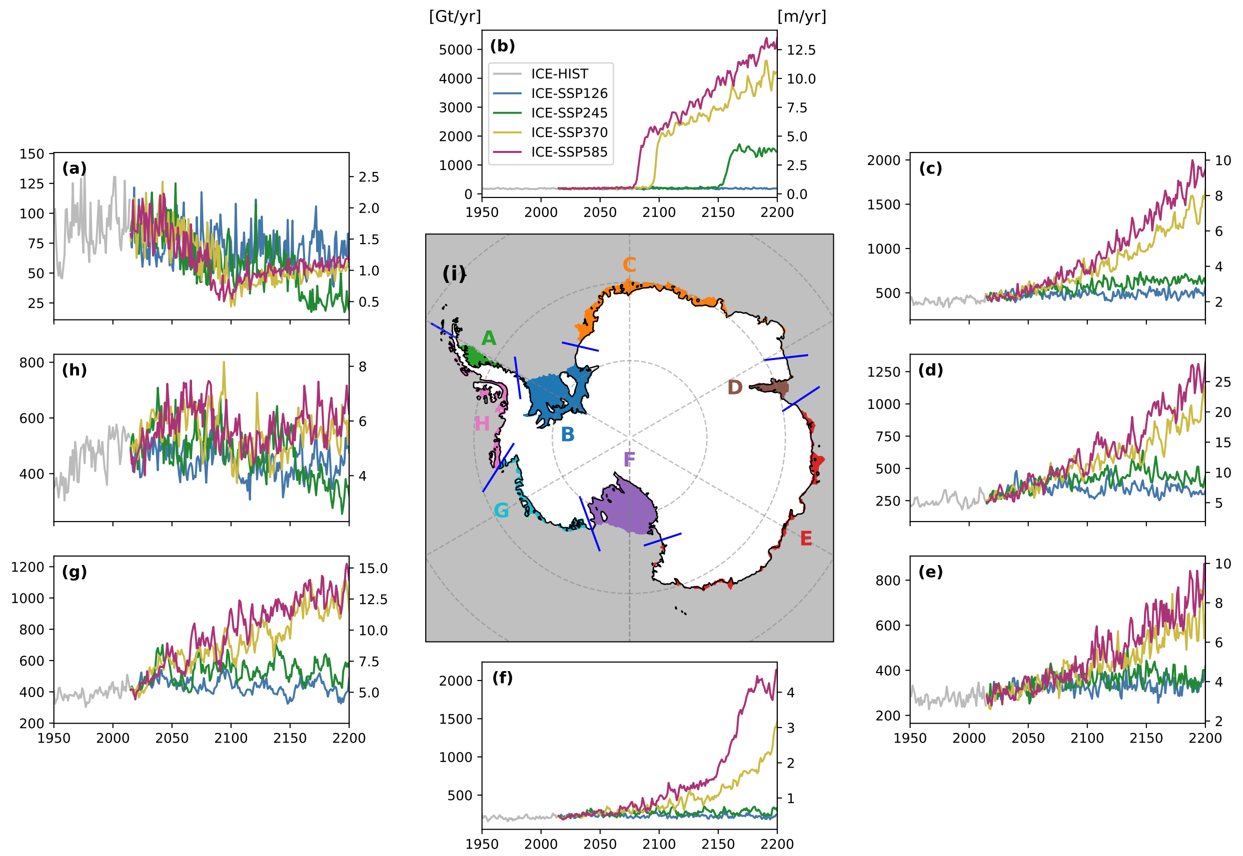

Supplementary Fig. S1 Projections of Antarctic basal melt by different regions. We divide the Antarctic ice shelves into eight regions: (a) the Larsen Ice Shelf; (b) the Filchner–Ronne Ice Shelf; (c) the Eastern Weddell region; (d) the Amery Ice Shelf; (e) the Australian sector; (f) the Ross Ice Shelf; (g) the Amundsen Sea; (h) the Bellingshausen Sea. Each panel displays area-integrated and area-averaged basal melt rates in units of Gt/yr (left scale) and m/yr (right scale), respectively. As the legend of panel (b) shows, line colours distinguish the HIST and SSP runs. Note that results from 1850 to 1950 are not shown.

Ensemble simulation results

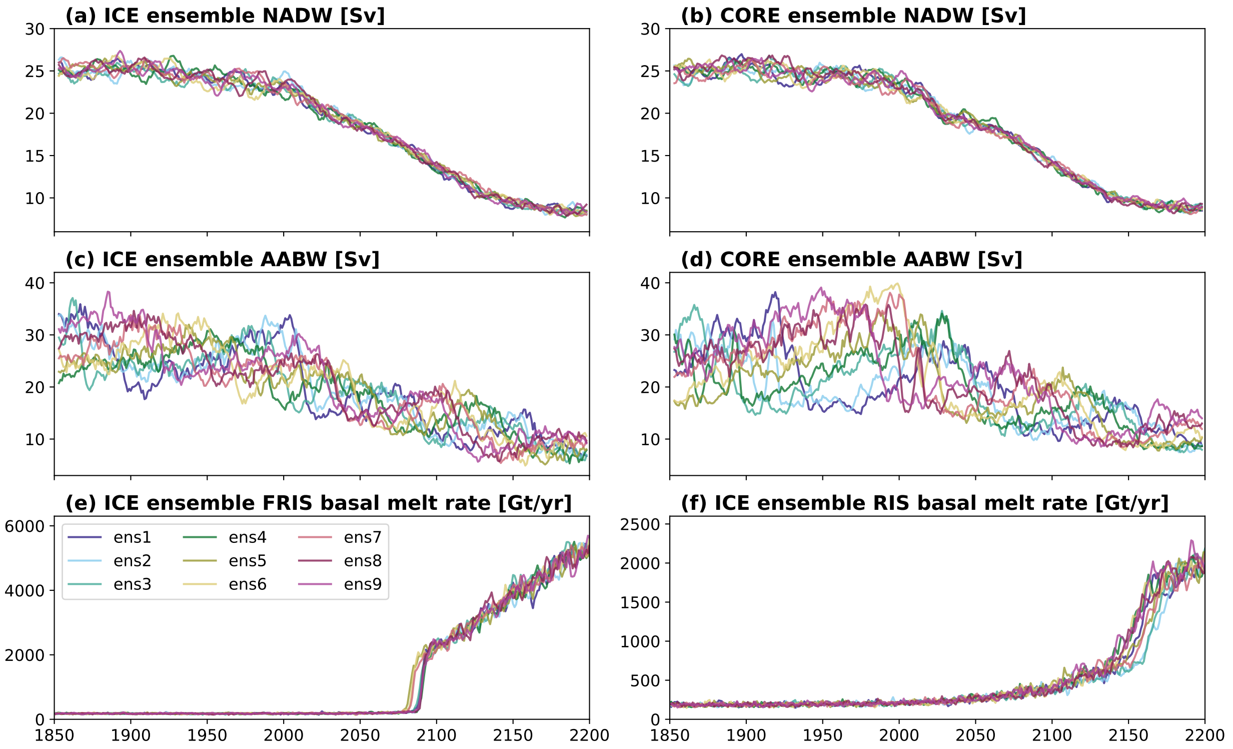

Supplementary Fig. S2 Results from ICE and CORE ensemble simulations. (a,b) NADW formation in all HIST-to-SSP585 ensemble members for ICE and CORE runs; (c,d) AABW formation in all HIST-to-SSP585 ensemble members for ICE and CORE runs; (e,f) Basal melt rate for FRIS and RIS in the HIST-to-SSP585 ensemble runs.

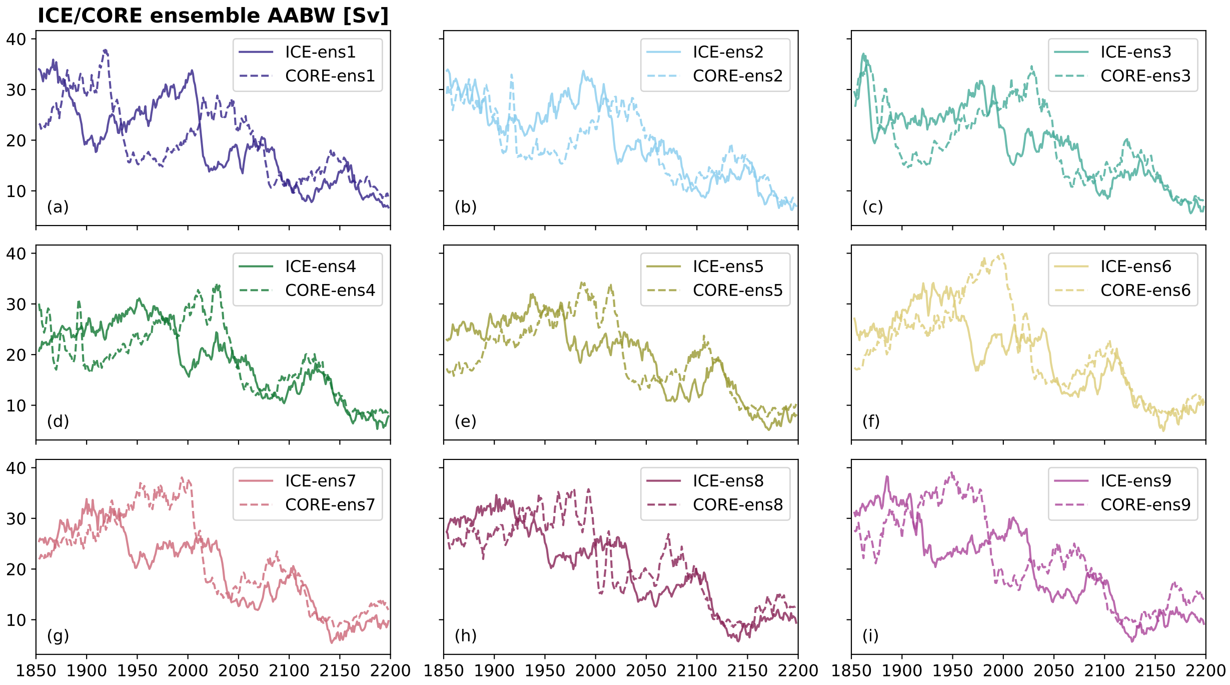

Supplementary Fig. S3 Individual plots for AABW formation in all HIST-to-SSP585 ensemble members. Solid lines and dashed lines indicate results from ICE and CORE ensemble members, respectively. Ensemble members are characterised by different branch-off years from the spin-up runs.

Density framework diagnosis

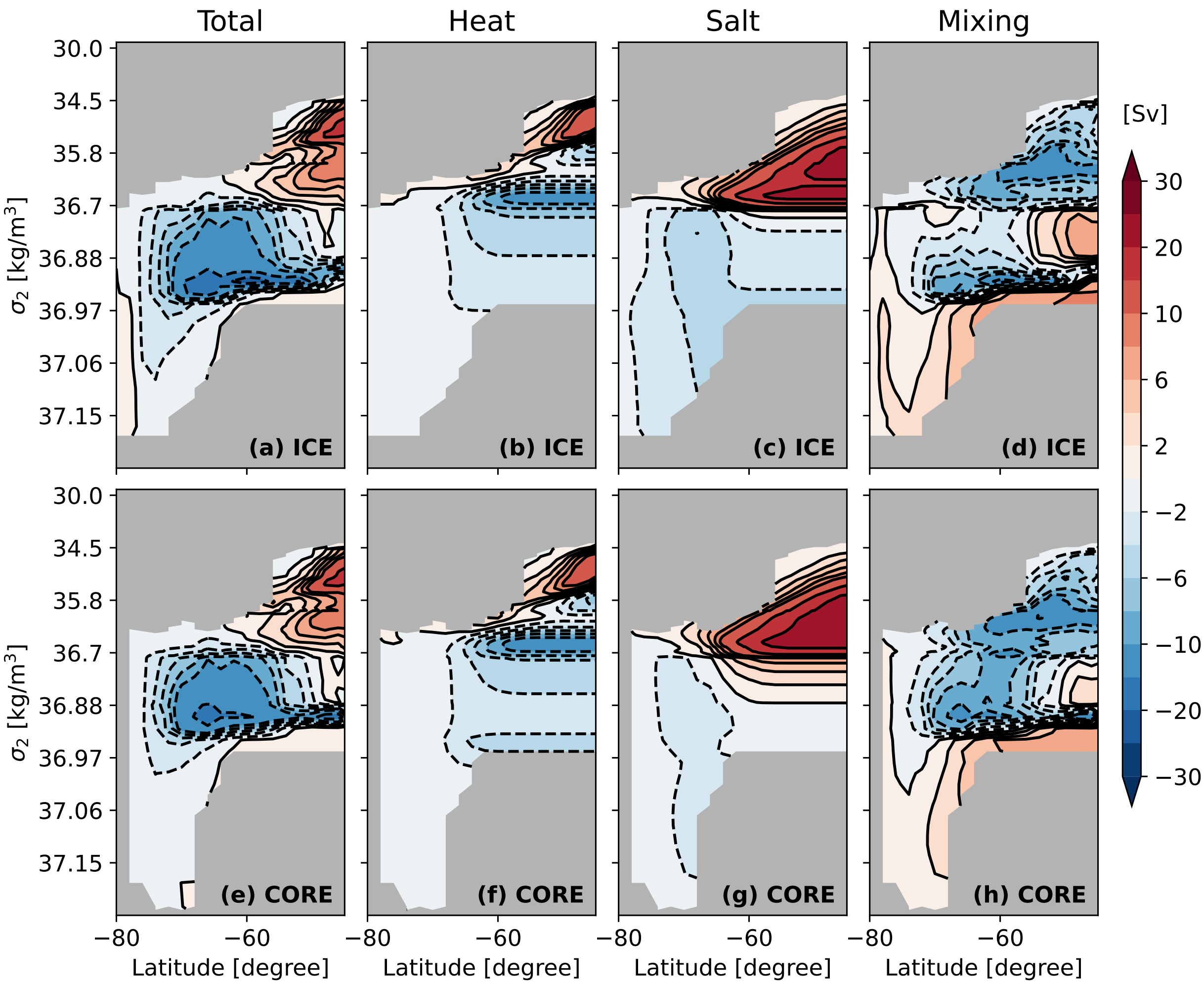

Supplementary Fig. S4 Decomposing density MOC in the Southern Ocean. (a,e) Density MOC from ICE-HIST and CORE-HIST. (b,f) Density MOC driven by surface water-mass transformation via heat flux. (c,g) Density MOC driven by surface water-mass transformation via salt flux. (d,h) Density MOC driven by internal water-mass transformation via ocean mixing. Red solid contours indicate clockwise circulation; blue dashed contours indicate counterclockwise circulation. The contours and the colourbar are not uniformly spaced. Density layers σ_2 represent potential densities referenced to 2000 m depth. Results are averaged from 1851 to 1899.

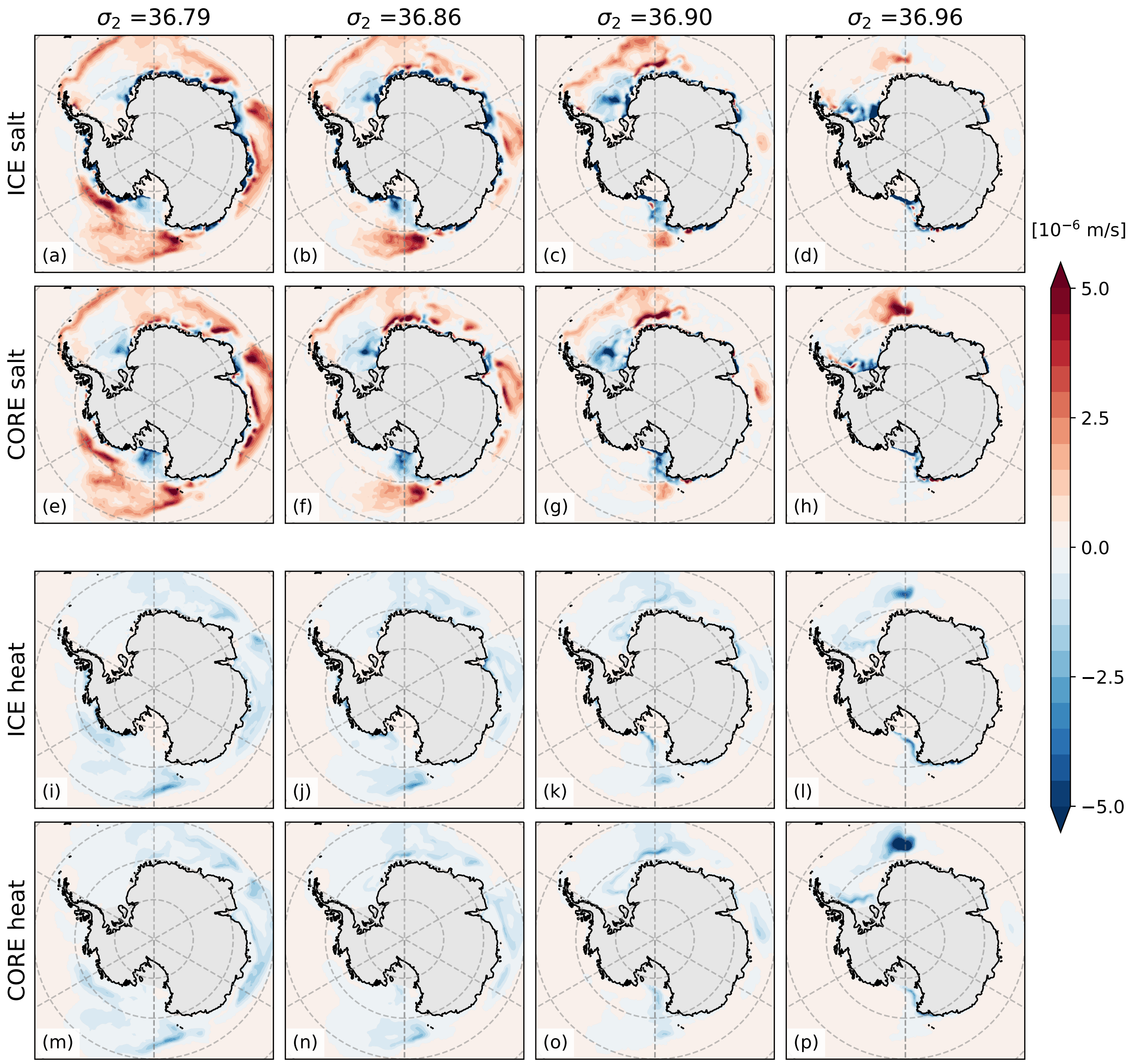

Supplementary Fig. S5 Surface water-mass transformation rates via salt and heat flux. (a–h) Water-mass transformation through salt flux in ICE-HIST (a–d) and CORE-HIST (e–h). Blue means density gain through brine rejection. (i–p) Water-mass transformation through heat flux in ICE-HIST (i–l) and CORE-HIST (m–p). Blue means density gain through surface cooling. Transformation rates at four individual density layers are shown here. Density layers σ_2 represent potential densities referenced to 2000 m depth. Results are averaged from 1851 to 1899.

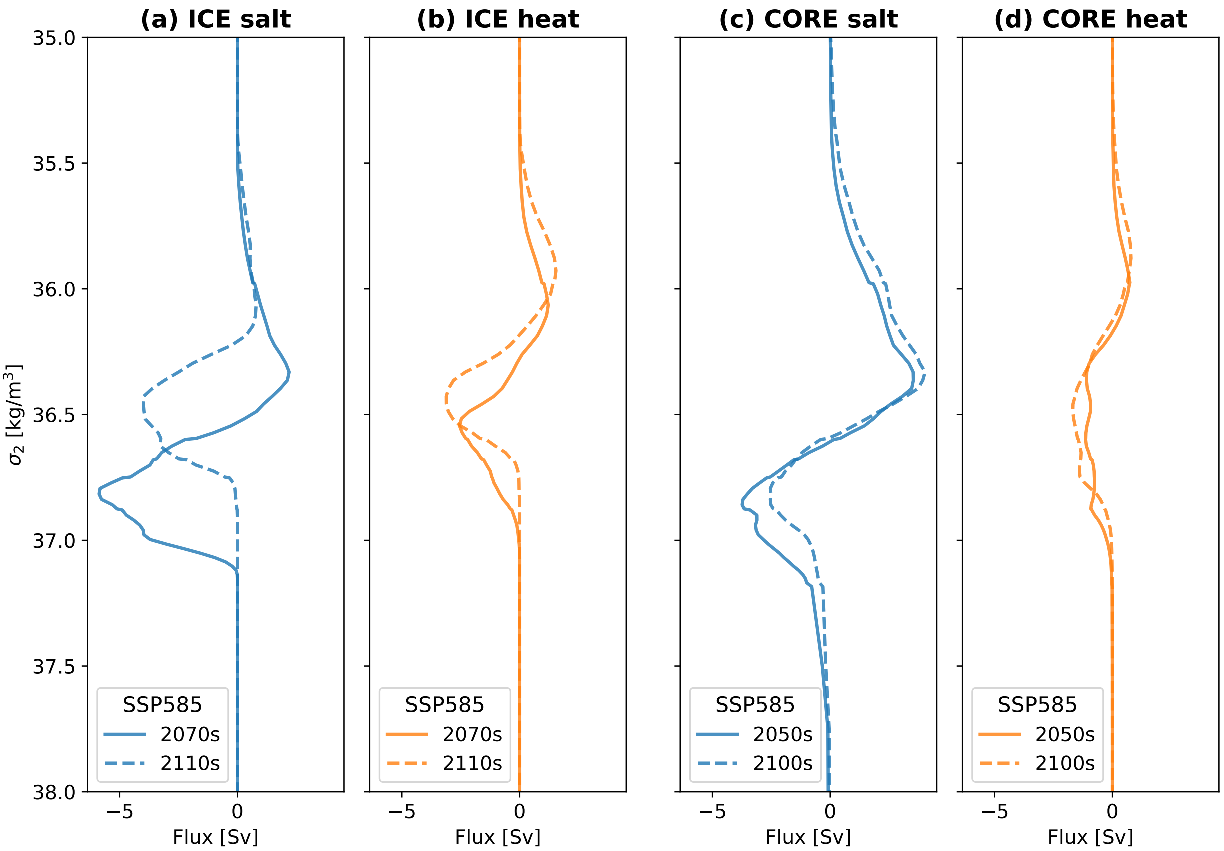

Supplementary Fig. S6 Evolution of surface water-mass transformation over the Antarctic continental shelf (shallower than 2000 m) in the SSP585 scenario. (a,b) Surface water-mass transformation via salt and heat flux in the 2070s and 2110s of ICE-SSP585. (c,d) Surface water-mass transformation via salt and heat flux in the 2050s and 2100s of CORE-SSP585. Note that negative transformation rates indicate dense-water formation through brine rejection or surface cooling processes. Density coordinate σ_2 denotes potential density referenced to 2000 m depth.

References for Supplementary Information

[1] Depoorter, M.A., Bamber, J.L., Griggs, J.A., Lenaerts, J.T.M., Ligtenberg, S.R.M., Broeke, M.R., Moholdt, G.: Calving fluxes and basal melt rates of Antarctic ice shelves. Nature **502**(7469), 89–92 (2013) https://doi.org/10.1038/nature12567

[2] Rignot, E., Jacobs, S., Mouginot, J., Scheuchl, B.: Ice-Shelf Melting Around Antarctica. Science **341**(6143), 266–270 (2013) https://doi.org/10.1126/science.1235798

[3] Adusumilli, S., Fricker, H.A., Medley, B., Padman, L., Siegfried, M.R.: Interannual variations in meltwater input to the Southern Ocean from Antarctic ice shelves. Nature Geoscience **13**(9), 616–620 (2020) https://doi.org/10.1038/s41561-020-0616-z

[4] Paolo, F.S., Gardner, A.S., Greene, C.A., Nilsson, J., Schodlok, M.P., Schlegel, N.-J., Fricker, H.A.: Widespread slowdown in thinning rates of West Antarctic ice shelves. The Cryosphere **17**(8), 3409–3433 (2023) https://doi.org/10.5194/tc-17-3409-2023

[5] Hellmer, H.H.: Impact of Antarctic ice shelf basal melting on sea ice and deep ocean properties. Geophysical Research Letters **31**(10), 1–4 (2004) https://doi.org/10.1029/2004GL019506

[6] Timmermann, R., Wang, Q., Hellmer, H.H.: Ice-shelf basal melting in a global finite-element sea-ice/ice-shelf/ocean model. Annals of Glaciology **53**(60), 303–314 (2012) https://doi.org/10.3189/2012AoG60A156

[7] Kusahara, K., Hasumi, H.: Modeling Antarctic ice shelf responses to future climate changes and impacts on the ocean. Journal of Geophysical Research: Oceans **118**(5), 2454–2475 (2013) https://doi.org/10.1002/jgrc.20166

[8] Schodlok, M.P., Menemenlis, D., Rignot, E.J.: Ice shelf basal melt rates around Antarctica from simulations and observations. Journal of Geophysical Research: Oceans **121**(2), 1085–1109 (2016) https://doi.org/10.1002/2015JC011117

[9] Mathiot, P., Jenkins, A., Harris, C., Madec, G.: Explicit representation and parametrised impacts of under ice shelf seas in the z^* coordinate ocean model NEMO 3.6. Geoscientific Model Development **10**(7), 2849–2874 (2017) https://doi.org/10.5194/gmd-10-2849-2017

[10] Naughten, K.A., Meissner, K.J., Galton-Fenzi, B.K., England, M.H., Timmermann, R., Hellmer, H.H., Hattermann, T., Debernard, J.B.: Intercomparison of Antarctic ice-shelf, ocean, and sea-ice interactions simulated by MetROMS-iceshelf and FESOM 1.4. Geoscientific Model Development **11**(4), 1257–1292 (2018) https://doi.org/10.5194/gmd-11-1257-2018

[11] Siahaan, A., Smith, R.S., Holland, P.R., Jenkins, A., Gregory, J.M., Lee, V., Mathiot, P., Payne, A.J., Ridley, J.K., Jones, C.G.: The Antarctic contribution to 21st-century sea-level rise predicted by the UK Earth System Model with an interactive ice sheet. The Cryosphere **16**(10), 4053–4086 (2022) https://doi.org/10.5194/tc-16-4053-2022

[12] Verfaillie, D., Pelletier, C., Goosse, H., Jourdain, N.C., Bull, C.Y.S., Dalaiden, Q., Favier, V., Fichefet, T., Wille, J.D.: The circum-Antarctic ice-shelves respond to a more positive Southern Annular Mode with regionally varied melting. Communications Earth & Environment **3**(1), 139 (2022) https://doi.org/10.1038/s43247-022-00458-x
